# Experimental—FEM Study on Effect of Tribological Load Conditions on Wear Resistance of Three-Component High-Strength Solid-Lubricant PI-Based Composites

**DOI:** 10.3390/polym13162837

**Published:** 2021-08-23

**Authors:** Sergey V. Panin, Jiangkun Luo, Dmitry G. Buslovich, Vladislav O. Alexenko, Lyudmila A. Kornienko, Svetlana A. Bochkareva, Anton V. Byakov

**Affiliations:** 1 Laboratory of Mechanics of Polymer Composite Materials, Institute of Strength Physics and Materials Science of Siberian Branch of Russian Academy of Sciences, 634055 Tomsk, Russia; buslovichdg@gmail.com (D.G.B.); vl.aleksenko@mail.ru (V.O.A.); rosmc@ispms.ru (L.A.K.); svetlanab7@yandex.ru (S.A.B.); bjakov@ispms.ru (A.V.B.); 2 Department of Materials Science, Engineering School of Advanced Manufacturing Technologies, National Research Tomsk Polytechnic University, 634050 Tomsk, Russia; jiangkun169@gmail.com

**Keywords:** polyimide, carbon fiber, polytetrafluoroethylene, graphite, molybdenum disulfide, elastic modulus, solid lubricant, wear resistance, porosity, roughness

## Abstract

The structure, mechanical and tribological properties of the polyimide-based composites reinforced with chopped carbon fibers (CCF) and loaded with solid-lubricant commercially available fillers of various natures were investigated. The metal- and ceramic counterparts were employed for tribological testing. Micron sized powders of PTFE, colloidal graphite and molybdenum disulfide were used for solid lubrication. It was shown that elastic modulus was enhanced by up to 2.5 times, while ultimate tensile strength was increased by up 1.5 times. The scheme and tribological loading conditions exerted the great effect on wear resistance of the composites. In the tribological tests by the ‘pin-on-disk’ scheme, wear rate decreased down to ~290 times for the metal-polymer tribological contact and to ~285 times for the ceramic-polymer one (compared to those for neat PI). In the tribological tests against the rougher counterpart (R*_a_*~0.2 μm, the ‘block-on-ring’ scheme) three-component composites with both graphite and MoS_2_ exhibited high wear resistance. Under the “block-on-ring” scheme, the possibility of the transfer film formation was minimized, since the large-area counterpart slid against the ‘non-renewable’ surface of the polymer composite (at a ‘shortage’ of solid lubricant particles). On the other hand, graphite and MoS_2_ particles served as reinforcing inclusions. Finally, numerical simulation of the tribological test according to the ‘block-on-ring’ scheme was carried out. Within the framework of the implemented model, the counterpart roughness level exerted the significantly greater effect on wear rate in contrast to the porosity.

## 1. Introduction

Polyimides (PI) are high-tech plastics with excellent mechanical properties, including at elevated temperatures. This enables their use for manufacturing polymer composites [1,2,3,4,5,6]. Various grades of the PI thermoplastics [7,8] are widely applied in electronics (as a substrate for microprocessors and liquid crystal displays) and medicine (for controlled drug delivery), as well as in the automotive (for the manufacturing of sealing rings and thrust washers) and aerospace (as parts operated at high temperatures and pressures, including electrical insulators) industries [9,10]. However, neat PI is rarely used for the fabrication of parts for tribological units, since it is characterized by a rather high wear rate under dry sliding friction conditions [11,12].

To solve this issue, a number of solid lubricant fillers are loaded for reducing its wear rate and the friction coefficient in tribological contacts. The most common ones are polytetrafluoroethylene (PTFE) [13,14], graphite (Gr) [15], molybdenum disulfide (MoS_2_) [16,17,18], etc.

To date, some research results have been published [19,20,21,22] on designing antifriction PI-based composites with low friction coefficients and improved wear resistance due to loading with PTFE, SiO_2_ and WS_2_ nanoparticles, etc. A partial decrease in mechanical properties is compensated by loading with reinforcing fibers, primarily carbon or glass ones [23,24]. Their adhesion to the polymer matrixes is mostly enhanced by treatment with coupling agents (for example, deposition of hydroxyl carbon nanotubes on the carbon fiber (CF) surfaces [25,26]).

Loading neat PI with reinforcing fibers and solid lubricant particles is an effective way to design antifriction composites [27,28]. Such materials are intended for operation under dry friction conditions. In this case, fibers improve both strength and wear resistance of the composites, while the solid lubricant component decreases the friction coefficient and the intensity of deformation processes on sliding surfaces [29].

In [30], the PI/PTFE/5%Gr composite has been studied (hereinafter, all percentages by weight unless otherwise indicated). It was shown that loading with these fillers provides the lowest friction coefficient and wear rate. The authors of paper [29] investigated wear rates of PI-based composites loaded with aramid fibers, Gr, MoS_2_, or PTFE under reciprocating friction motion. In this case, the Gr showed the best lubricity at low friction coefficients and wear rates. It should be noted that both friction and wear processes depend both on material structure and test methods, applied loads, sliding speeds, temperatures and lubrication conditions [31].

We investigated the mechanical and tribological characteristics of two- and three-component PI-based composites loaded with PTFE and chopped CF (CCF, 200 µm in length) at different load-speed (P·V) modes according to the ‘pin-on-disk’ scheme [32]. The PI/10%PTFE/10% (annealed) CF composite possessed the greatest wear resistance. Its wear rate decreased by ~310 times for metal-polymer tribological contacts and by ~285 times for ceramic-polymer ones. At the same time, loading PI with PTFE reduced the elastic modulus and worsened the structure homogeneity (due to the uneven distribution of PTFE in the bulk material). Thus, it is relevant to load CCF several millimeters long for improving the mechanical properties (the elastic modulus and tensile strength) of the PI-based composites.

However, tribological testing according to the ‘pin-on-disk’ and “block-on-ring” scheme possess some particular features. Among them are: (i) counterpart roughness and specific heat, (ii) load-speed conditions, (iii) heat release rate, etc. In addition, loading with various solid lubricant fillers affects the structure formation in different ways. Thus, mechanical and tribological properties change as well. In doing so, the composites with highest tribological performance under particular testing conditions might be less efficient at their variation. The latter was studied in details with the use of Finite Element Method, when composites’ porosity as well as counterpart roughness were varied.

Thus, a comparison of various types of commercially available solid lubricant fillers is relevant. In addition, tribological test conditions (including loading patterns and counterpart roughness) can have a decisive effect on their wear resistance.

The purpose of this research was to design high-strength antifriction PI-based composites loaded with solid lubricant fillers of various natures (organic PTFE, inorganic Gr and MoS_2_) as well as reinforced with CCF for applications in various tribological conditions. For this purpose, their structures and wear rates after tests according to the ‘pin-on-disk’ and ‘block-on-ring’ schemes with different roughness of counterparts were compared. The first scheme is more often employed in the scientific literature, while the second conditions are closer to industrial friction units (seals, thrust bearings, etc.).

The second section describes materials and research methods. Section 3.1 contains data on the structure, physical and mechanical properties of the studied composites. Section 3.2 is devoted to the analysis of the tribological characteristics obtained under sliding against metal and ceramic counterparts according to the ‘pin-on-disk’ scheme. Section 3.3 includes the test results under various ‘block-on-ring’ loading conditions. These patterns, including an analysis of the scratch test results, are discussed in Section 4. Section 5 presents the results of numerical simulation of the effects identified in the experimental investigation, which precedes the final conclusions.

## 2. Materials and Methods

### 2.1. Materials

The ‘Solver PI-Powder 1600’ powder (Solver, Jiande City, Zhejiang Province, China) with an average particle size of 16 μm was used. In addition, the following fillers were loaded: (i) the ‘Fluralit’ fine powder with an average diameter of less than 3 μm obtained from the ‘F-4’ fluoroplastic thermal decomposition (‘Fluralit synthesis’ LLC, Moscow, Russia), (ii) molybdenum disulfide MoS_2_ (Climax Molybdenum Co, Fort Madison, IA, USA, ∅1–7 μm), and (iii) colloidal graphite (∅1–4 μm). In addition, CCFs (Tenax^®^-A, Teijin Carbon Europe Gmbh, Wuppertal, Germany) with a length of 1–2 mm (aspect ratio ~ 100) were used as reinforcing fibers. To remove a coupling (technological) agent, initially contained on the CCF surfaces (for epoxy binders), they were annealed in air using a ‘Memmert UF 55’ oven (Binder, Tuttlingen, Germany) at a temperature of 500 °C for 30 min.

### 2.2. Fabrication of the PI-Based Composites

The polymer powder and fillers were mixed by dispersing the suspension components in alcohol using a ‘PSB-Gals 1335-05’ ultrasonic cleaner (‘PSB-Gals’ Ultrasonic equipment center, Moscow, Russia). Processing time was 3 min; generator frequency was 22 kHz. After mixing, a suspension of the components was dried in an oven with forced ventilation for 3 h at a temperature of 120 °C. PI-based composites were fabricated by hot pressing at a pressure of 15 MPa and a temperature of 370 °C with a subsequent cooling rate of 2 °C/min.

Table 1 shows compositions of the studied PI-based composites (in volume and weight percentages).

### 2.3. Physical and Mechanical Properties

**Figure 1:**
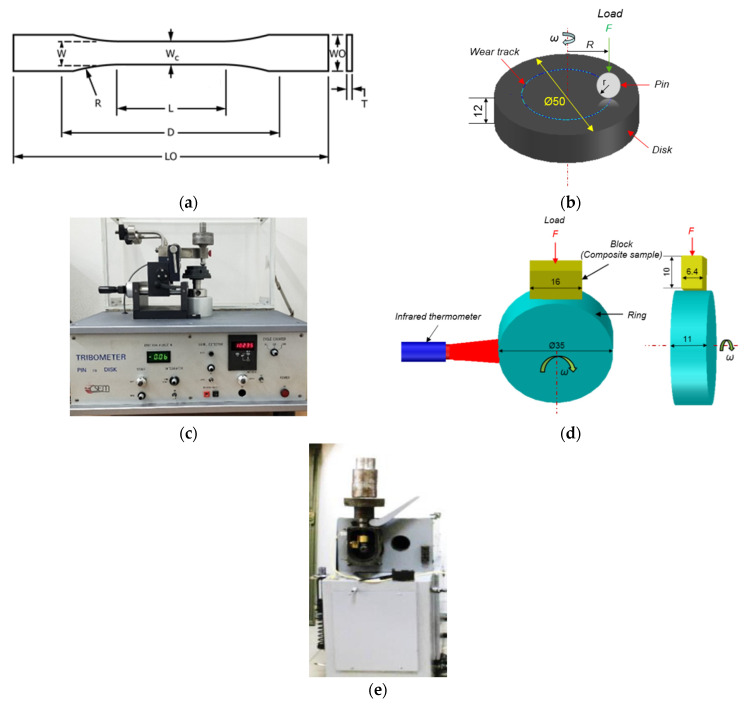
Dimension of a dog-bone specimen (**a**) and the schematic representation of the tribological testing schemes and equipment: “pin-on-disk” (**b**) and CSEM CH2000 tribometer (**c**); “block-on-ring” (**d**) and 2070 SMT-1 friction testing machine (**e**).

Tensile properties of the ‘dog-bone’ shaped PI-based samples (Figure 1a) were measured under tension using an ‘Instron 5582’ electromechanical testing machine (Instron, Norwood, MA, USA). The number of the samples of each type was at least four. The following samples’ dimensions were taken: T = 3.2 ± 0.4 mm; W = 3.18 ± 0.5 mm; L = 9.53 ± 0.5 mm; WO = 9.53 + 3.18 mm; LO = 63.5 ± 0.4 mm; D = 25.4 ± 5 mm; R = 12.7 ± 1 mm. The samples were stretched until the break. The strain was measured with the use of an extensometer.

### 2.4. Tribological Characteristics

Dry sliding friction tests were carried out according to the ‘pin-on-disk’ scheme (Figure 1b) at a load (*P*) of 5 N and a sliding speed (*V*) of 0.3 m/s. A ‘CSEM CH-2000’ tribometer (CSEM) was used (Figure 1c) in accordance with ASTM G99. The maximum Hertzian contact pressure (*P_max_*) was 417.5 MPa. The tribological tests were conducted using metal and ceramic counterparts (balls made of bearing steel (60 HRC) and ZrO_2_, respectively). The counterpart diameters were 6 mm; their surface roughness was R*_a_* = 0.02 µm (it was assessed with the help of a ‘New View 6200’ optical interferential profilometer, ‘Zygo Corporation’, Middlefield, CT, USA). The testing distance was 1 km and the tribological track radius was 16 mm.

In addition, wear resistance was evaluated according to the ‘block-on-ring’ scheme (Figure 1d) using a ‘2070 SMT-1’ friction testing machine (Figure 1e, Tochpribor Production Association, Ivanovo, Russia). Load (*P*) on the samples ranged 60–180 N, while sliding speed (*V*) values were varied from 0.1 up to 0.5 m/s. The block’s dimension was equal to: length 16 mm × width 6.4 mm × height 10 mm. The maximum Hertzian contact pressure (*P_max_*) levels were 66.9, 88.3, 102.1, and 115.8 MPa, respectively. A steel counterpart was made of the outer ring of a commercial bear. It had a disk shape with a diameter of 35 mm and a width of 11 mm. The counterpart surface roughness was R*_a_* = 0.20–0.25 µm (it was evaluated with the ‘New View 6200’ profilometer). The counterpart temperature was assessed using a ‘CEM DT-820’ non-contact infrared (IR) thermometer (Shenzhen Everbest Machinery Industry Co., Ltd., Shenzhen, China). The friction coefficient was continuously recorded by an on-line data acquisition system attached to the tester. The measured friction coefficient was the average value throughout the test, excluding running-in period. Wear rates were determined by measuring width and depth of friction tracks according to stylus profilometry, followed by multiplication by its length. The wear rate levels were calculated taking into account the load and sliding distance values:

(1) $$\mathrm{Wear}\;\mathrm{rate}\;=\frac{\mathrm{volume}\;\mathrm{loss}\;\left({\mathrm{mm}}^{3}\right)}{\mathrm{load}\;\left(N\right)\times \mathrm{sliding}\;\mathrm{distance}\;\left(m\right)}$$

### 2.5. Temperature Measurement

Figure 1d shows a schematic representation of the temperature measurement process on the counterpart surface during the tribological tests (according to the ‘block-on-ring’ scheme). A ‘CJMCU-MLX90614ESF-DCI’ module with an ‘MLX90614 Melexis’ infrared thermometer (Microelectronic Integrated System, Ypres, Belgium) was used as a non-contact sensor. The sensor has a measurement accuracy of 0.5 °C (at a resolution of 0.02 °C); its field of view was 5°. The reported values were the average temperatures of all objects in the sensor’s field of view.

### 2.6. Scratch Tests

The scratch tests were performed using a ‘Revetest RST’ setup (CSM Instruments, Needham, MA, USA). A spherical indenter had the cone shape for a Rockwell hardness tester with angles of 120° and a curvature radius of 200 μm. Two conditions were applied: (a) increasing load from 0.5 up to 10 N; (b) at a constant load of 5 N. The indenter travelling distance was 5 mm at a speed of 40 mm/min. The friction force and the penetration depth of the indenter were estimated. The friction coefficient was calculated using the formula:

μ (Scratch Friction Coefficient) = Friction Force/Normal Load(2)

### 2.7. Structural Studies

The surface topography of the friction tracks was studied using a ‘Neophot 2’ optical microscope (Carl Zeiss, Jena, Germany) equipped with a ‘Canon EOS 550D’ digital camera (Canon Inc., Tokyo, Japan) and an ‘Alpha-Step IQ’ contact profilometer (KLA-Tencor, Milpitas, CA, USA).

The cleaved surfaces of the notched specimens mechanically fractured after exposure in liquid nitrogen were used for structure studies. A ‘LEO EVO 50’ scanning electron microscope (Carl Zeiss, Oberkochen, Germany) with an ‘Oxford INCA X-Max80’ unit for EDS microanalysis (Oxford Instruments, Abingdon, Oxfordshire, UK) was employed at an accelerating voltage of 20 kV.

## 3. Results and Discussion

### 3.1. Structure, Physical and Mechanical Properties

**Figure 2:**
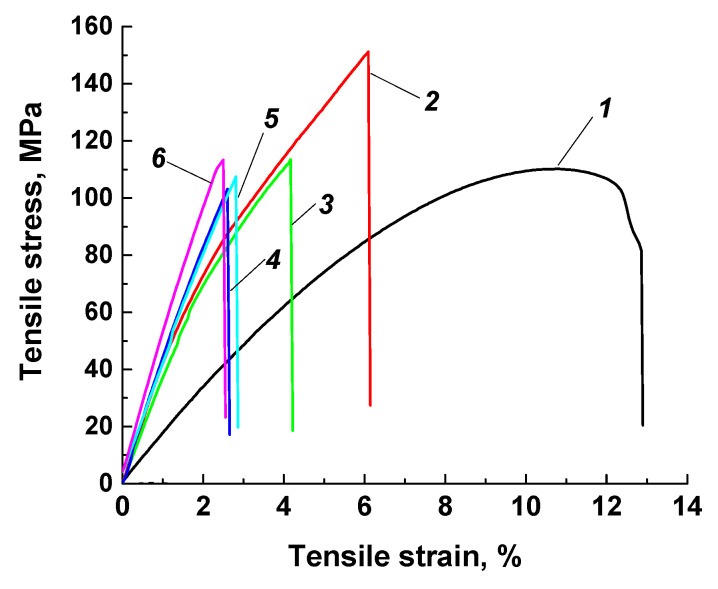
The strain–stress diagrams for neat PI (1), as well as the PI/10CCF (2), PI/10CCF/10PTFE (3), PI/10CCF/10Gr (4), PI/10CCF/10MoS_2_ (5) and PI/10CCF/23MoS_2_ (6) composites.

Figure 2 and Table 2 show the physical and mechanical properties of the fabricated PI-based composites. Note that the content of MoS_2_ particles varied due to the fact that its density was approximately two times higher than that of other solid lubricant fillers. Therefore, the equality of it’s either the weight (10 wt. %) or volume (23 wt. %) content was ensured.

The key PI mechanical properties improved markedly after loading with 10% CCF: the elastic modulus was increased by 2.5 times and ultimate tensile strength was enhanced by 1.5 times. Subsequent addition of solid lubricant particles to the ‘PI + CCF’ mixture slightly reduced the deformation and strength characteristics (with the exception of elongation at break).

**Figure 3:**
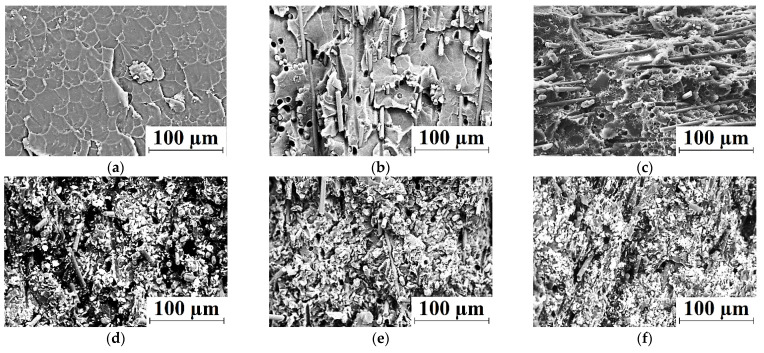
SEM-micrographs illustrating the structure for neat PI (**a**), as well as the PI/10CCF (**b**), PI/10CCF/10PTFE (**c**), PI/10CCF/10MoS_2_ (**d**), PI/10CCF/10Gr (**e**), and PI/10CCF/23MoS_2_ (**f**) composites.

Figure 3 presents SEM-micrographs of both neat PI and PI-based composite structures. In neat PI, a uniform cellular-like structure was formed (the ‘cell’ sizes were about 15 µm), which approximately corresponded to the dimensions of the initial powder particles. After loading with CCF, the ‘cell’ sizes did not change or decreased slightly. Reinforcing fibers were distributed rather evenly without preferential orientation (Figure 3b). Note that CCFs were well wetted by the polymer matrix. Additional loading with ‘Fluralit’ particles was accompanied by the formation of a significant number of pores of various diameters (less than 10 μm, Figure 3c). We suggest that this was due to the presence of air on the polymer filler surfaces, which was released in the bulk material upon hot pressing and remained inside. However, this did not affect the strength properties of the composites, which was confirmed by the above mechanical test data.

Loading with 10% Gr or MoS_2_ inorganic solid lubricants markedly changed the polymer structure (Figure 3d,e). Firstly, these fillers did not melt at the hot-pressing temperature. Secondly, their sizes were comparable to those of the PI powder that ensured their uniform distribution in the CCF reinforced polymer matrix. Thirdly, they contributed to a more uniform sintering of the three-component PI-based composite due to their high thermal conductivity. As a result, a heterogeneous polymer composite was formed, reinforced with both CCF and finely dispersed inclusions. However, a formed structure of the PI/10CCF/10PTFE composite also ensured a satisfactory level of its mechanical properties (Figure 2).

Additional loading of the ‘PI + CCF’ mixture with a larger (by weight) amount of MoS_2_ particles (23 wt. % or 6.6 vol. %, Table 1) caused an “excessive” content of filler particles (Figure 3f). Probably, this solved the issue of reducing the friction coefficient (described below), but was accompanied by the increase in the Shore D hardness, elastic modulus, and ultimate tensile strength. However, the value of elongation at break was reduced.

### 3.2. The Tribological Tests According to the ‘Pin-On-Disk’ Scheme

**Figure 4:**
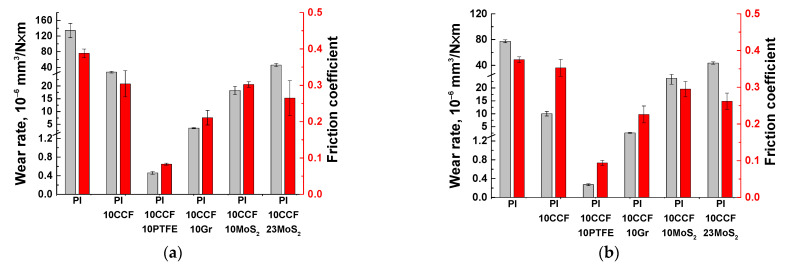
Wear rates and the friction coefficients for neat PI (1), as well as the PI/10CCF, PI/10CCF/10PTFE, PI/10CCF/10Gr, PI/10CCF/10MoS_2_, and PI/10CCF/23MoS_2_ composites. Metal (**a**) and ceramic (**b**) counterparts. The ‘pin-on-disk’ scheme.

**Figure 5:**
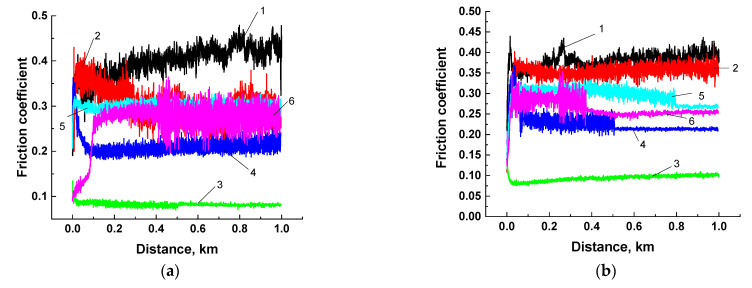
The friction coefficients for neat PI (1), as well as the PI/10CCF (2), PI/10CCF/10PTFE (3), PI/10CCF/10Gr (4), PI/10CCF/10MoS_2_ (5), PI/10CCF/23MoS_2_ (6) composites. Metal (**a**) and ceramic (**b**) counterparts. The ‘pin-on-disk’ scheme.

Next, the tribological properties of the three-component PI-based composites were analyzed under sliding against the metal and ceramic counterparts (the ‘pin-on-disk’ scheme, Table 3; Figure 4 and Figure 5a,b). Since the balls from commercial bearings were used, their surface roughness R*_a_* was typical and did not exceed ~0.02 μm.

The lowest both wear rate and friction coefficient were observed for the PI/10CCF/10PTFE composite: wear rate decreased by ~291 times for the metal- and by ~286 times for the ceramic-polymer tribological contacts. Loading with inorganic solid-lubricant fillers (Gr, MoS_2_) improved wear resistance of the composites to a much lesser extent: by 37 and 30 times for graphite, and by 7 and 3 times for MoS_2_ in the metal- and ceramic-polymer tribological contacts, respectively. Note that changes in wear rates and the friction coefficients correlated fairly well with each other. This confirmed the solid lubricating effect under the given scheme and conditions of the tribological tests.

It is seen from Table 3 as well as Figure 4 and Figure 5 that loading with neat PI with 23 wt. % (6.6 vol. %) MoS_2_ caused an increase in wear rate relative to that for the content of 10 wt. % (although the friction coefficient decreased slightly). For this reason, a comparative analysis of the tribological characteristics of the composites loaded with various solid lubricant particles was carried out at their equal weight content of 10 wt. % (when tested according to the ‘block-on-ring’ scheme).

The aspect of improving wear resistance of high-strength polymers was traditionally substantiated by the formation of transfer films on counterparts, which transformed the tribological pair into a ‘polymer-polymer’ one [33]. In addition, the discussion of wear-related results was carried out in terms of the formation of secondary structures on the friction surfaces of polymers and composites. The latter were formed from both debris and oxidized particles of the counterpart materials [34]. In these studies, the analysis of micrographs of the friction/wear surfaces of all studied samples and counterparts was carried out according to the same principle.

**Figure 6:**
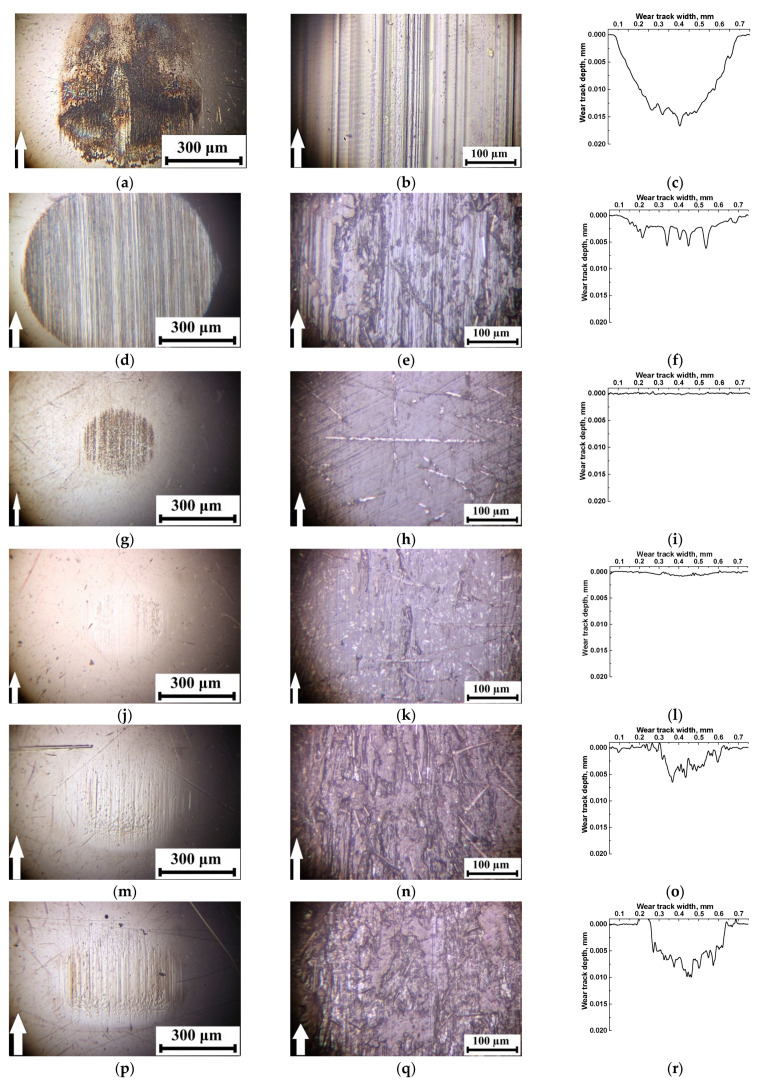
The optical images of the friction surfaces on the metal counterpart and neat PI (**a**–**c**), as well as the PI/10CCF (**d**–**f**), PI/10CCF/10PTFE (**g**–**i**), PI/10CCF/10Gr (**j**–**l**), PI/10CCF/10MoS_2_ (**m**–**o**), and PI/10CCF/23MoS_2_ (**p**–**r**) composites.

Neat PI was characterized by rapid wear, which was accompanied by the formation of deep (up to 0.017 mm) grooves and polymer debris attached to the metal counterpart surface (including development of tribological oxidation, Figure 6a–c). High hardness of the polymer did not enable to resist wear due to the sliding counterpart action [35,36]. In addition to longitudinal grooves, ‘row’ structures (tens of microns wide) were formed on the neat PI friction surface across the sliding direction at a distance not exceeding 10–20 μm (Figure 6b; left).

Loading with reinforcing CCF slightly reduced wear rate of the polymer composite (Figure 6f), but they exerted a ‘cutting’ effect on the counterpart surface (Figure 6d). On the other hand, secondary structures in the form of irregularly-shaped debris islands were formed and adhered on the friction surface (Figure 6e).

Subsequent addition of 10% PTFE in the PI/10CCF composite significantly decreased wear rate, which was accompanied by the formation of a transfer film on the counterpart and an almost smooth surface of the wear track on the sample (Figure 6g–i): only thin abrasive paper traces were observed on the surface (Figure 6h). According to the authors, the entire friction track surface was covered with a thin PTFE layer, which caused a multiple decrease in the friction coefficient.

Loading the PI/10CCF composite with graphite was accompanied by enhancing both wear rate and the friction coefficient (Figure 6l). The friction track was quite smooth on this composite, while a subtle transfer film was visible on the steel counterpart surface (Figure 6j). The low wear rate level minimized the possibility of the formation of secondary structures on it (Figure 6k) [27,35].

At loading with MoS_2_ particles wear rates increased regardless of their content (Figure 6o,r). This resulted in the formation of secondary structures on the composite friction surface (Figure 6n,q). On the other hand, a thick transfer layer was formed on the counterpart surface due to the MoS_2_ chemical activity (namely, the ability to oxidize). It contained particles of the transferred polymer composite in the form of a ‘loose’ layer with pores and micro-grooves (Figure 6m,p). Note that no wear traces were observed on the steel counterpart surface after loading the composites with all three types of solid lubricant particles.

**Figure 7:**
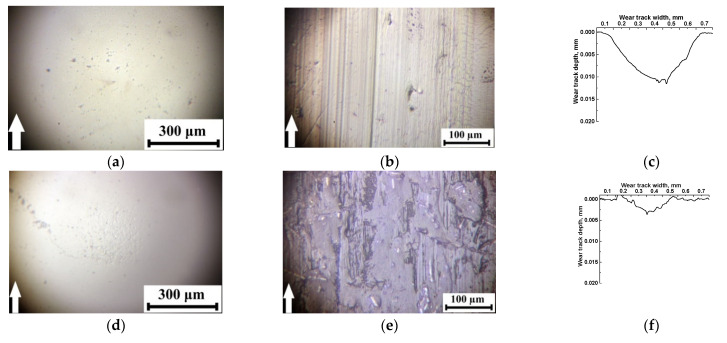

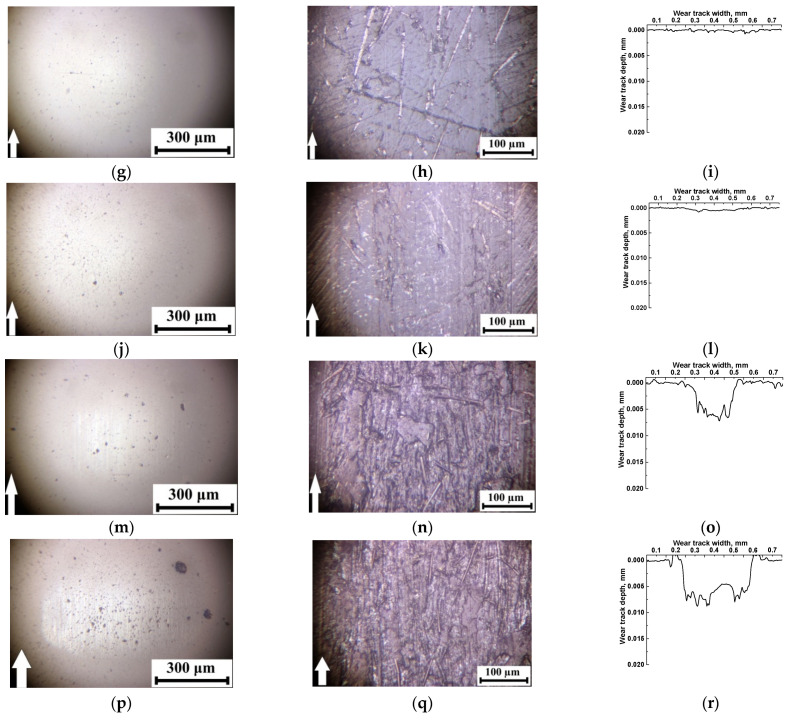
The optical images of the friction surfaces on the ceramic counterpart and neat PI (**a**–**c**), as well as the PI/10CCF (**d**–**f**), PI/10CCF/10PTFE (**g**–**i)**, PI/10CCF/10Gr (**j**–**l**), PI/10CCF/10MoS_2_ (**m**–**o**), and PI/10CCF/23MoS_2_ (**p**–**r**) composites.

In the ceramic-polymer contact (Figure 7), a similar pattern of the wear tracks was observed on all composite and counterpart surfaces. The only difference was the impossibility to adhere the transfer film (formed from debris) on the inert ceramic counterpart. It is suggested that reducing wear rate (in comparison with that for neat PI) was mainly associated with the formation of secondary structures on the friction surfaces on the composites (primarily in the form of films from solid lubricant fillers). This reduced the friction coefficients and improved wear resistance (Table 3). The most effective solid lubricant filler was PTFE as well. Note that the friction coefficients of the composites with different solid lubricant fillers were similar in both types of the tribological contacts (Figure 7 and Table 3).

**Figure 8:**
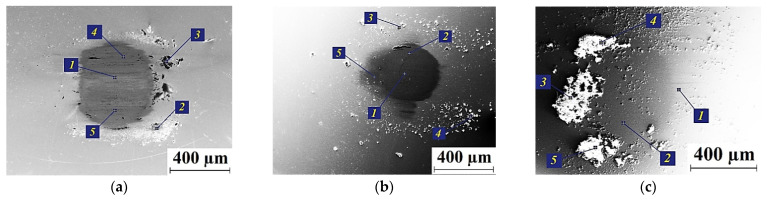
SEM micrographs of the steel counterpart surfaces after the tribological tests: the PI/10CCF/10PTFE (**a**), PI/10CCF/10Gr (**b**), and PI/10CCF/10MoS_2_ (**c**) composites.

Since the formation of the transfer films affected wear rates of the composites under the used test scheme, the EDS microanalysis of the metal counterpart friction surfaces was carried out (Figure 8). It was concluded that both debris and transfer films were on the steel counterpart surfaces after friction on the CCF-reinforced PI-based composites filled with all solid lubricant particles. Their composition is shown in Table 4.

Fluorine (which was a part of fluoroplastic) and secondary tribological oxidation products were found in the transfer film on the steel counterpart for the PI/10CCF/10PTFE composite (points 4 and 5; Figure 8a). The fluoroplastic content was higher in debris outside the transfer film (point 3). A similar pattern was observed for the PI/10CCF/10Gr composite. Carbon in the amount of several weight percent was included in the transfer film (points 2 and 3; Figure 8b), but its content was significantly higher outside it (point 4).

The transfer film had low contrast on the counterpart for the MoS_2_-contained composite (Figure 8c). At the same time, sulfur (as a component of MoS_2_) was not detected (point 2). However, the sulfur content reached up to 20 wt. % (points 3, 4 and 5) as a part of numerous rather large debris “islands” (including due to agglomeration).

Thus, PTFE was the most effective solid lubricant filler for PI in the tribological tests on the smooth counterpart according to the ‘pin-on-disk’ scheme (*P* = 5 N; *V* = 0.3 m/s). In this case, the uniform transfer film was formed on the counterpart friction surface. A similar solid lubricant fluoroplastic-containing film was revealed on the friction surface of the CCF-reinforced PI-based composite. These data correlated with similar conclusions published by other authors, for example, for PEEK-based composites [37,38].

Since the designed CCF-reinforced PI-based composites should operate under various conditions (loads, speeds, temperatures, roughness of mating pairs, etc.), their tribological characteristics were also investigated on the metal counterpart according to the ‘block-on-ring’ scheme. The outer ring of a commercial steel bearing (60 HRC) was taken as a counterpart. Its roughness R*_a_* was 0.25 μm (grade 9), which was an order of magnitude higher than that for the steel ball (R*_a_* = 0.02 μm, grade 13). In addition, there was the difference in specific pressure and the pattern of heat dissipation (heating was less for the larger steel ring due to better heat dissipation). The steel counterpart was used precisely because of the higher reactivity compared to Al_2_O_3_ ceramics. Parametric studies were carried out with varying the P·V tribological modes.

### 3.3. Tests on the Metal Counterpart (the ‘Block-On-Ring’ Scheme)

**Figure 9:**
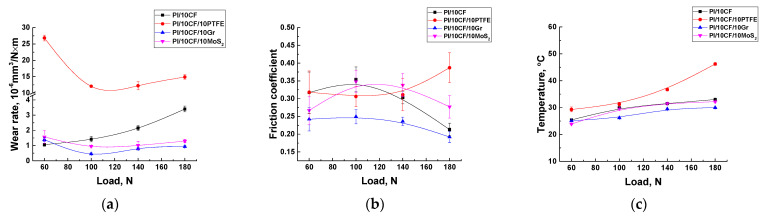
Wear rates (**a**), the friction coefficients (**b**) and the counterpart temperatures (**с**) on the friction surfaces of the PI-based composites.

Figure 9 shows the tribological properties of the PI-based composites (including the counterpart temperature) tested in the load (*P*) range of 60–180 N at the constant sliding speed (*V*) of 0.3 m/s. The maximum Hertzian contact pressure values were estimated: 66.9 MPa for *P* = 60 N; 86.3 MPa for *P* = 100 N; 102.1 MPa for *P* = 140 N; and 115.8 MPa for *P* = 180 N. Regarding the ‘pin-on-disk’ scheme, a fundamentally different pattern of the influence of the solid lubricant fillers on the tribological characteristics of the composites was observed.

At the minimum load of 60 N, the lowest wear rate of ~1.06 × 10^−6^ mm^3^/N×m was for the CCF-reinforced PI-based composites. A slightly higher wear rate was typical for the composites additionally loaded with Gr and MoS_2_. A highest wear rate of ~26.8 × 10^−6^ mm^3^/N×m was found for the one loaded with PTFE.

At *P* = 180 N, wear rate of the PI/10CF composite gradually enlarged up to ~2.13 × 10^−6^ mm^3^/N×m (as the load increased). This parameter varied slightly (0.45–1.57 × 10^−6^ mm^3^/N×m) over the entire range of the applied loads for both PI/10CF/10MoS_2_ and PI/10CF/10Gr ones. In the case of the PI/10CF/10PTFE composite, wear rate first decreased and then rose up to 14.9 × 10^−6^ mm^3^/N×m. Thus, there was an ‘inversion’ in the trend of the effect of the solid lubricants on wear resistance relative to the ‘pin-on-disk’ scheme.

In addition, in contrast to the ‘pin-on-disk’ tests there was a different trend in the change in the friction coefficients. First of all, under lower contact pressure (*P_max_* = 66.9–115.8 MPa compared to 417.5 MPa for the ‘pin-on-disk’ scheme), the friction coefficients were noticeably higher than those for the ‘pin-on-disk’ scheme. In particular, the minimum value of 0.24 was typical for the composite loaded with graphite (at the load of 60 N, *P*_max_ = 66.9 MPa), while it was maximum (0.318) for the PI/10CCF/10PTFE sample. At the same time, this difference was noticeably lower in absolute value than for the ‘pin-on-disk’ scheme. There were also two interesting trends: as the load increased, the friction coefficient of the graphite-contained composite gradually decreased down to 0.192, while on the contrary, it enhanced up to 0.387 for the one loaded with PTFE. Therefore, the comparability of the values and the rather high level of the friction coefficients for both composites loaded with solid lubricant particles and without them testified for their weak effect on wear resistance.

Note, the counterpart temperature varied in different ways with increasing load from 60 up to 180 N. As well to a certain extent it correlated with the trend of the changes in wear rates. For all composites except the PI/10CCF/10PTFE one, it almost linearly enhanced with increasing load, and did not exceed 33 °C at *P* = 180 N. The exception was the PTFE loaded composite; the counterpart temperature was higher than 29.3 °С even at the minimum load, while it was maximum (46.2 °С) at *P* = 180 N. Note that the measured temperature did not exactly correspond to those at the contact spots, but it could be used for the qualitative interpretation of the obtained results.

**Figure 10:**
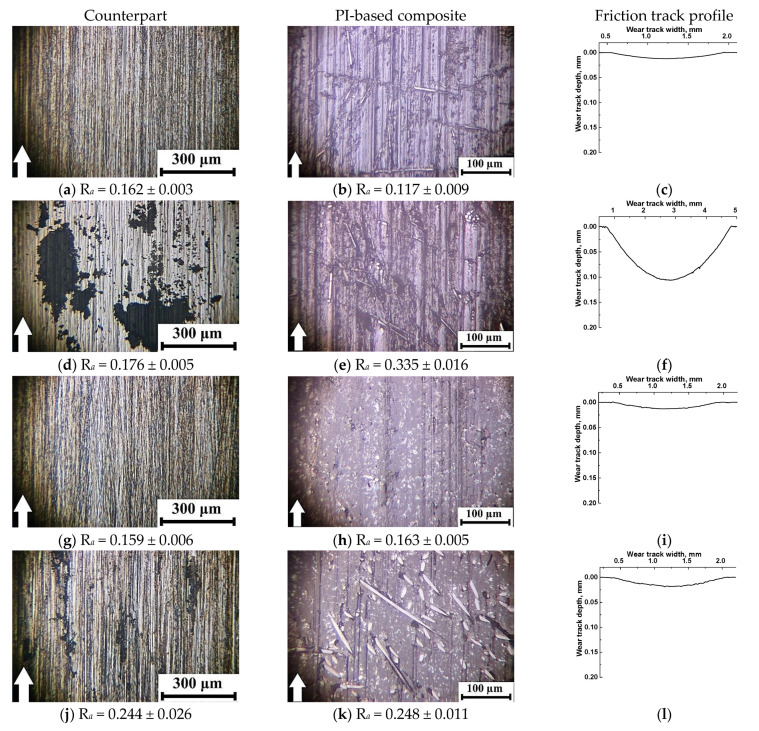
The micrographs of the wear tracks on the metal counterpart and the PI/10CCF (**a**–**c**), PI/10CCF/10PTFE (**d**–**f**), PI/10CCF/10Gr (**g**–**i**), and PI/10CCF/10MoS_2_ (**j**–**l**) composites. The ‘mild’ mode (*P* = 60 N; *V* = 0.3 m/s).

**Figure 11:**
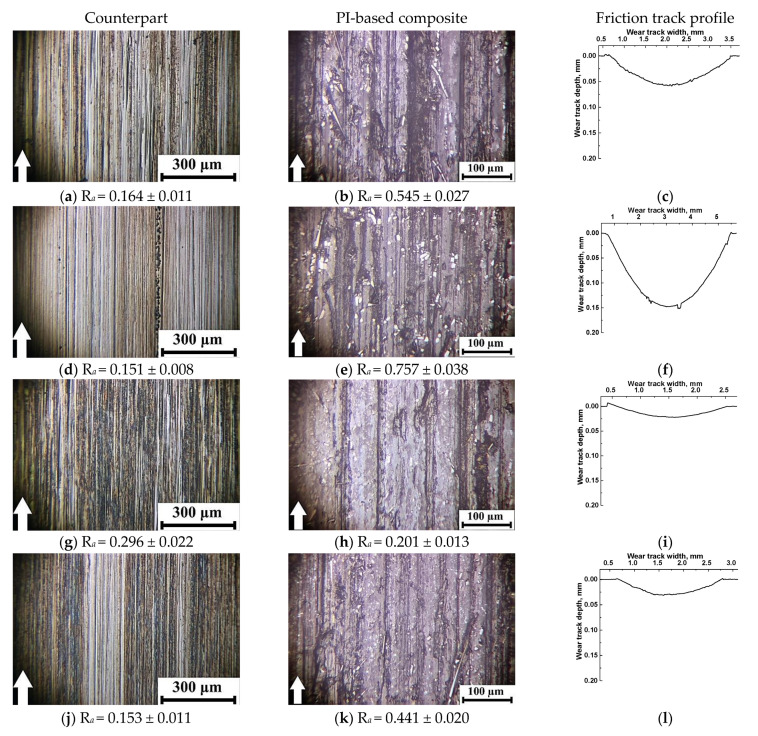
The micrographs of the wear tracks on the metal counterpart and the PI/10CCF (**a**–**c**), PI/10CCF/10PTFE (**d**–**f**), PI/10CCF/10Gr (**g**–**i**), and PI/10CCF/10MoS_2_ (**j**–**l**) composites. The ‘severe’ mode (*P* = 180 N; *V* = 0.3 m/s).

In this way, the change in the scheme and conditions of the tribological tests radically varied the characteristics of the studied three-component composites (first of all, their wear resistance). For a better understanding of the reasons for the observed effects, Figure 10 and Figure 11 show the wear track surfaces of the samples after the tribological test under ‘mild’ and ‘severe’ modes. They are briefly analyzed below. As a part of the analysis, the authors paid special attention to the surface roughness on both composites and counterparts, whose were indicated next to the micrographs.

Under the ‘mild’ tribological loading conditions (*P* = 60 N; *V* = 0.3 m/s; Figure 10), wear rates were comparable for all composites (with the exception of the PI/10CCF/10PTFE one and did not exceed ~1.57 × 10^−6^ mm^3^/N×m). At the same time, roughness of the counterpart surfaces changed insignificantly in the range of 0.159–0.175 µm, which was slightly lower than that for the initial level of ~0.20 µm. It increased a little bit up to 0.244 μm only for the PI/10CCF/10MoS_2_ composite. In the cases of the PI/10CCF/10PTFE (Figure 10d) and PI/10CCF/10MoS_2_ (Figure 10j) ones, debris in the form of thin dark flakes was observed on the counterpart surfaces. The reason for this fact could be an excessive volume of worn-out material.

The surface roughness on the friction tracks of the PI-based composites changed less predictably. Roughness was minimum (0.117 µm) on the PI/10CCF one (Figure 10b,c), which was noticeably lower than that for the counterpart. This could be due to ‘polishing’ of this hard-composite surface with the steel counterpart.

For the PI/10CCF/10PTFE composite (Figure 10d–f), wear rate increased by an order of magnitude up to ~26.8 × 10^−6^ mm^3^/N×m. Island-shaped secondary structures, caused by the excessive presence of debris, were observed on the wear track surface. In addition, signs of the material tearing were found despite the wear track profile was quite smooth. The reason could be the negative effect of PTFE on the structure formation. As a result, roughness significantly increased up to 0.335 µm, which was about twice that of the counterpart.

For the CCF-reinforced composites loaded with Gr and MoS_2_, wear rates were very low. At the same time, the ‘sawtooth’ variations (most likely associated with the protrusion of CCF above the surface) were visible on the wear track profilograms (Figure 10i,l). According to the optical micrographs of the wear track surfaces on the PI/10CCF/10Gr composite, it was very smooth. This corresponded to an R*_a_* value of 0.163 μm (Figure 10h) identical to the counterpart roughness (Figure 10g). Numerous CCF protruded above the friction surface were observed on the PI/10CCF/10MoS_2_ composite. The higher roughness of 0.248 µm on its wear track surface (Figure 10k) was close to the counterpart parameter of 0.244 µm (much like the previous case).

Under the ‘severe’ tribological loading conditions (*P* = 180 N; *V* = 0.3 m/s; Figure 11), the counterpart surface roughness of 0.15–0.16 μm was almost identical to those for the ‘mild’ ones. The only exception was the PI-based composite loaded with graphite (R*_a_* = 0.296 μm; Figure 11a,d,j). This result could be associated with the presence of debris on the counterpart surface in the form of a discontinuous film and the optical (interference) method of roughness assessing (Figure 11g). After the test of the PI/10CCF/10MoS_2_ composite, a similar film was observed on the steel counterpart surface; however, its roughness of 0.153 μm was close or slightly lower than the initial one.

The micrographs of the wear tracks on the PI-based composites were more informative. Much like the ‘mild’ tribological test conditions, roughness of these surfaces was proportional to their wear rates. In the case of the PI/10CCF composite, longitudinal grooves oriented in the sliding direction were evident on the wear track surface (Figure 11b), which was reflected in its profile (Figure 11c). Most likely, such grooves were formed from non-adhered debris. Roughness on the composite wear track surface of 0.545 μm was several times higher than that on the counterpart.

In the case of the PI/10CCF/10PTFE composite (with the maximum wear rate, Figure 11f), CCF were intensely fractured. The wear track surface was also damaged as a result of plowing with debris (Figure 11e). Roughness multiplied up to 0.757 μm and was the highest among all the studied materials.

In the PI/10CCF/10Gr composite, characterized by the minimal wear rate under the ‘severe’ tribological test conditions, the friction track surface was the smoothest (Figure 11i). It contained only individual longitudinal grooves, while its surface roughness 0.201 µm was also minimal (Figure 11h).

The minimum wear rate was as well found for the PI/10CCF/10MoS_2_ composite, similar to the one loaded with graphite. In this case, however, the wear track surface was more damaged by longitudinal grooves (Figure 11k,l), while its roughness of 0.441 µm was twice as high as that for the PI/10CCF/10Gr sample and three times higher than that for the steel counterpart.

**Figure 12:**
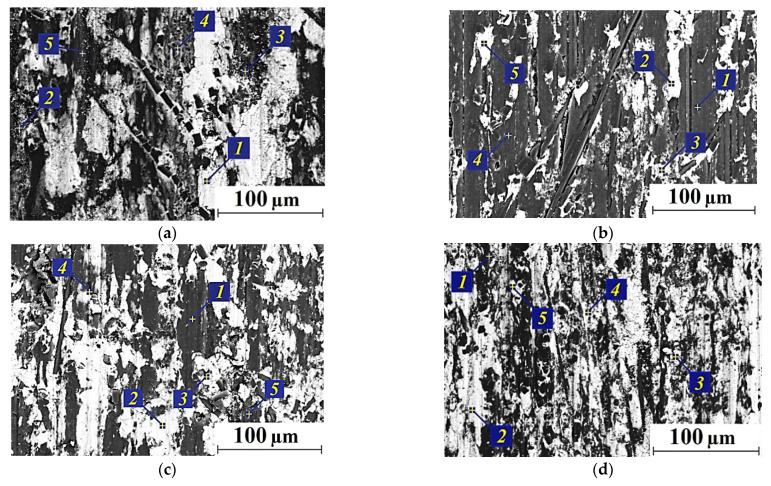
The optical micrographs of the wear tracks on the PI/10CCF (**a**), PI/10CCF/10PTFE (**b**), PI/10CCF/10Gr (**c**), and PI/10CCF/10MoS_2_ (**d**) composites. The ‘severe’ mode (*P* = 180 N, *V* = 0.3 m/s).

Since wear rates were associated with the formation of secondary structures on the wear track surfaces, further studies were carried out using the EDS data (Figure 12, Table 5). The obtained results indicated that the tribological oxidation processes were decisive for the mentioned surface modification. This was confirmed, first of all, by the enhanced oxygen content. An increase in the load resulted in the intensification of the oxidation processes (including due to rising the counterpart temperature) and the formation of more uniform secondary structure layers. The latter could serve as the surface wear protection. The presence of iron and chromium was due to their transfer from the steel counterpart surface.

**Figure 13:**
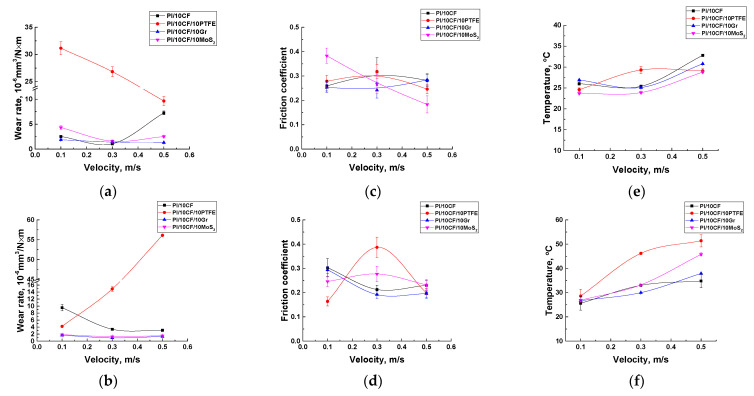
Wear rates (**a**,**b**), the friction coefficients (**c**,**d**), and the counterpart temperature (**e**,**f**) for the PI-based composites vs. sliding speed: (**a**,**c**,**e**)—*P* = 60 N; (**b**,**d**,**f**)—*P* = 180 N.

Further, the effect of sliding speed on the tribological characteristics of the PI-based composites was investigated for the ‘block-on-ring’ scheme (Figure 13).

Analysis of the results enabled to conclude that the PI/10CCF/10PTFE composite was characterized by the worst wear resistance. At the lowest applied load of 60 N, its greatest difference from other composites was observed at the minimum sliding speed of 0.1 m/s, while the variation was almost leveled at the maximum *V* = 0.5 m/s (Figure 13a). Note that the pattern of the friction coefficient variation versus the sliding speed did not have the same trend (Figure 13c), i.e., the kinetics of the friction coefficient and wear rate were weakly correlated. An even less obvious trend was evident for the sliding speed versus temperature dependence (Figure 13e). The counterpart temperature did not exceed 26.9 °C at *V* = 0.1 m/s and practically did not change at 0.3 m/s. The exception was the PI/10CF/10PTFE composite (*T* = 29.4 °C). On the contrary, it increased for all composites, except for the mentioned one, but to a rather small extent (*T* = 28.8–32.8 °C). Thus, the effect of the counterpart temperature on the development of the tribological processes at the lowest load of 60 N could be considered insignificant.

At the maximum applied load of 180 N, wear rate of the PI/10CCF/10PTFE composite enhanced sharply with an increase in sliding speed: its value was 56.1 × 10^−6^ mm^3^/N×m at *V* = 0.5 m/s (Figure 13b). Vice versa, it decreased for all other composites, including for the one without solid lubricant particles. The nature of the friction coefficients variation with increasing sliding speed was generally the same as for wear rates of most composites. The PI/10CCF/10PTFE was the only exception. Typically, the counterpart temperature enhanced with sliding speed increasing. However, it was minimal for the PI/10CCF and PI/10CCF/10Gr composites and had the maximum for the PI/10CCF/10PTFE sample (Figure 13f).

Thereby, the PI/10CCF/10PTFE composite showed the lowest tribological characteristics when tested according to the ‘block-on-ring’ scheme. This was not associated with the friction coefficient, as well as the counterpart temperature. Possible reasons are discussed in the next section.

## 4. Discussion

The ‘inverse’ effect of PTFE on wear resistance during the tribological tests according to both ‘pin-on-disk’ and ‘block-on-ring’ schemes is discussed below as the most unexpected result.

**Figure 14:**
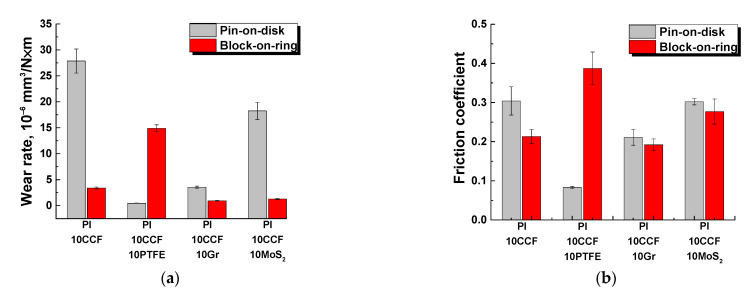
Wear rates (**a**) and the friction coefficients (**b**) for the PI/10CCF, PI/10CCF/10PTFE, PI/10CCF/10Gr, and PI/10CCF/10MoS_2_ composites at *P* = 5 N and *V* = 0.3 m/s for the ‘pin-on-disc’ scheme as well as *P* = 180 N and *V* = 0.3 m/s for the ‘block-on-ring’ one.

In the case of the smoother counterpart (the roughness was R*_a_* of 0.02 μm) in the tribological tests according to the ‘pin-on-disk’ scheme, the change in wear rates and the friction coefficients correlated with each other (Figure 14a,b).

The authors believe that the reason was the low counterpart roughness and the possibility of its facilitated sliding against the surface of the solid-lubricant composite. This was the characteristic of the metal counterpart, although it was well traceable for the ceramic one. In this case, the presence of PTFE particles, to a certain extent, formed the more defective structure (with microporous) that did not reduce its wear resistance. Moreover, solid lubricant particles were located along the entire sliding path in this tribological test scheme, which also contributed to the renewal of the transfer film on the counterpart. However, this effect was not observed under the ‘block-on-ring’ scheme, when the limited amount of solid lubricant particles on the counterpart friction track was not enough to form a transfer film over this large area [29,33]. Probably, the issue could be solved by increasing the content of solid lubricant particles to tens of weight percent. However, this would severely embrittle the polymer composites.

The second important factor was the fact that fine (units of microns) both MoS_2_ and graphite particles ‘bound’ well to the polymer matrix. This agrees well with the literature data [39,40] as well as with our previous results [32]. As a result, they performed both roles of dispersed strengthening particles and solid lubricant inclusions. However, they were tighter bonded to the polymer matrix in contrast to PTFE. For this reason, solid lubrication function of the former was realized to a lesser extent.

For the ‘block-on-ring’ scheme, the friction coefficient was noticeably higher (over 0.2). However, it did not differ much for all studied PI-based composites both with solid lubricant inclusions and CCF-reinforced two-component one (Figure 14b). Highly likely, the reason was the higher counterpart roughness. Therefore, the key reason for the wear processes developed in the solid-lubricant PI-based composites was no less specific pressure or heat dissipation. Namely, it was the difference in roughness of the counterparts for both applied tribological test schemes.

It was of importance that the counterpart temperatures were almost the same at the load of 60 N for all solid-lubricant composites and the CCF-reinforced one. Note that solid lubricant inclusions practically did not fulfill this functional role. Moreover, wear rates almost did not increase for the PI-based composites loaded with Gr and MoS_2_ with enhancing loads. Also, a very significant temperature rise of the counterpart was at the maximum load of 180 N (Figure 13f). This confirmed that PTFE did not serve as a solid lubricant. According to the authors, great frictional heating of the PI/10CF/10PTFE composite could be associated with its inhomogeneous (more porous) structure.

In this way, the above results indicated that solid lubricating inclusions had a non-decisive (minimal) effect on wear resistance under the studied conditions of the tribological tests (the ‘block-on-ring’ scheme and, above all, the counterpart roughness). To a greater extent, the role of such inclusions was associated with the structure modification of the CCF-reinforced polymer matrix.

**Figure 15:**
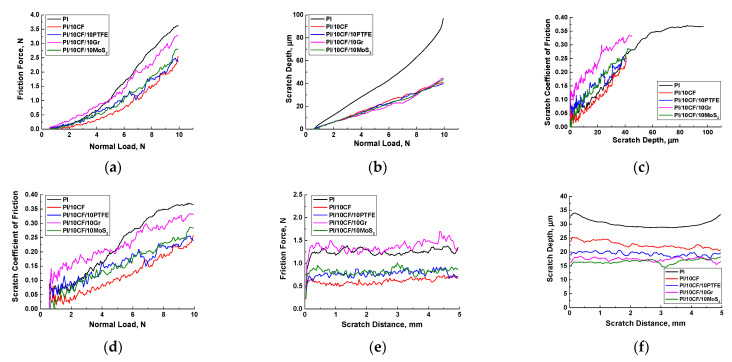
The scratch test results for the CCF-reinforced PI-based composites: increasing (**a**–**d**) and constant (**e**,**f**) loads.

**Figure 16:**
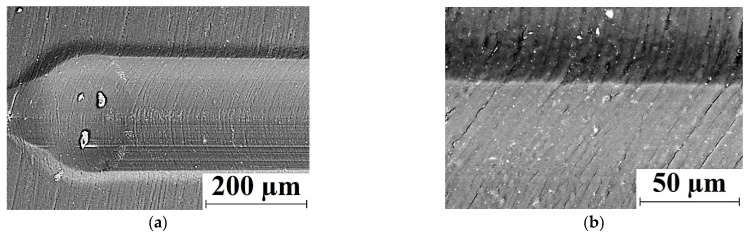

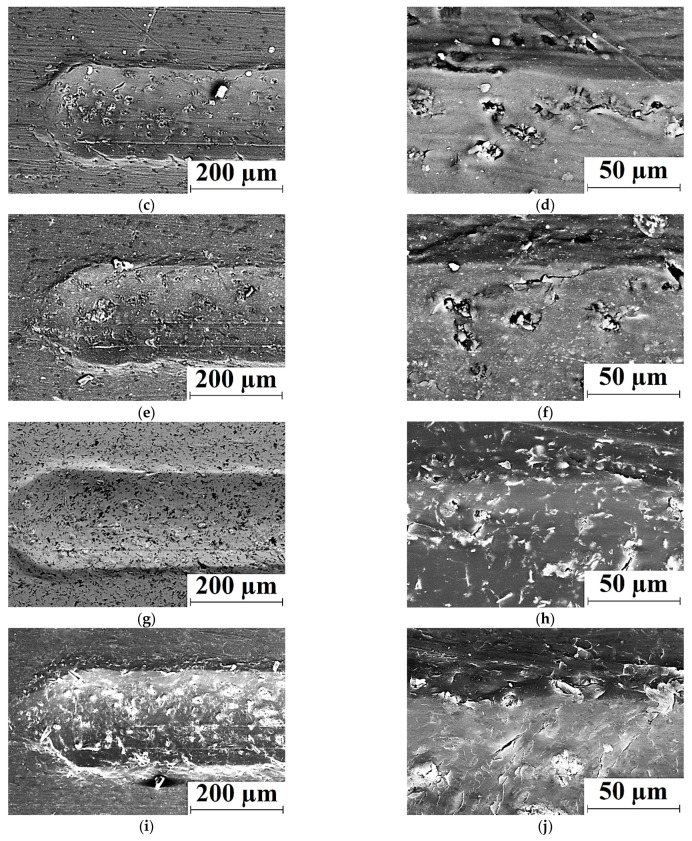
SEM-micrographs of the PI (**a**,**b**), PI/10CCF (**c**,**d**), PI/10CCF/10PTFE (**e**,**f**), PI/10CCF/10Gr (**g**,**h**), and PI/10CCF/10MoS_2_ (**i**,**j**) composites after the scratch tests; increasing load.

Since the ‘block-on-ring’ tribological test with the higher counterpart roughness could be associated, to some extent, with multiple ‘plowing’ effects of microprotrusions on the steel counterpart surface, the scratch test was performed [41]. The loading conditions were varied: (i) with increasing load from 0.5 up to 10 N; and (ii) at the constant load of 5 N. The results are presented in Figure 15. SEM micrographs of the scratch tracks formed under increasing load are shown in Figure 16.

### 4.1. Scratch Tests with Increasing Load

Figure 15a shows the dependence of the friction force versus the applied load. The best resistance to the ‘plowing’ action of the indenter was provided by neat PI and the PI/10CCF/10Gr composite. In the case of neat PI, this could be due to the greater effect of plastic deformation associated with the indenter penetration into the polymer (Figure 15b). This was also confirmed by the formation of more pronounced plastic deformation at the scratch track edge (Figure 16a–c). On the other hand, the curve for the two component PI/10CCF composite was located below those for all other ones, including the PI/10CCF/10PTFE and PI/10CCF/10MoS_2_ samples. For these composites, the indenter movement could cause localized cracking within scratch-tracks (Figure 16i,o) due to the negative influence of the solid lubricants on the structure homogeneity. It could be assumed that the presence of graphite in the CCF-reinforced composite further increased its strength, preventing the easier ‘plowing’ movement of the indenter. Its interaction with filler particles was accompanied by cracking (Figure 16l), which could provide additional resistance to the movement. In addition, the PTFE, Gr and MoS_2_ particles were structure modifiers rather than facilitating the indenter (plowing) movement during the scratch test.

At the same time, the presence of PTFE and MoS_2_ particles in the PI/10CCF/10PTFE and PI/10CCF/10MoS_2_ composites did not greatly change resistance to the indenter ‘plowing’ movement (as in in the case of the PI/10CCF sample). Similar conclusions followed from the analysis of the graphs presented in Figure 15d. The only difference was that the friction coefficient of the indenter on neat PI increased nonlinearly as the load enhanced. At the same time, this parameter rose at approximately the same rate for all other composites.

The graph of the indenter penetration depth versus the applied load (Figure 15b), in particular, characterized hardness of the investigated materials. All CCF-reinforced composites behaved in a similar way (a linear increase in the analyzed dependence was observed). Also, the scratch track widths were noticeably narrower (Figure 16b,d,g,j,m) than that on the neat PI (Figure 16a). At the same time, the penetration depth, as expected, increased faster into neat PI, and the pattern of its enhancing was characterized as nonlinear.

The curves of the friction coefficient versus the indenter penetration depth are shown in Figure 15c. In general, all of them showed a similar trend, including quantitative characteristics. However, the curve for the PI/10CCF/10Gr composite was located above all the others, which again indicated its higher resistance to the indenter plowing.

Summarizing the obtained results, it was possible to conclude the following:

- The indenter (plowing) movement was easiest on the PI/10CCF composite.
- The presence of graphite additionally strengthened the PI/10CCF/10Gr composite and increased its resistance to the indenter (plowing) movement.
- Despite the formation of microporosity, resistance the indenter (plowing) movement on the PI/10CCF/10PTFE composite was similar to that for the PI/10CCF/10MoS_2_ one.

### 4.2. Scratch Tests at the Constant Load

The test results at the constant indenter load were generally consistent with those described above. Figure 15e shows the dependence of the friction force versus the indenter movement distance. As in Figure 15a, both neat PI and the PI/10CCF/10Gr composite provided similar resistance to the indenter movement and the PI/10CCF one possessed the lowest level.

Figure 15f presents the relationship between the indenter penetration depth and its movement distance. In this case, the trend changed little relative to the results of the scratch test under increasing loads. The maximum scratch track depth for neat PI was obviously associated with the absence of the CCF-reinforcement, while the position of the curve for the PI/10CCF composite was slightly unexpected. Note that the PI/10CCF/10PTFE composite showed resistance to the indenter movement comparable to that for both PI/10CCF/10Gr and PI/10CCF/10MoS_2_ ones. This was despite the extremely high wear rate upon the tribological tests according to the ‘block-on-ring’ scheme. The authors believe that the reason was the scratch effect locality and the indenter smoothness. This was similar to the low counterpart roughness (to a certain extent) in the tribological tests according to the ‘pin-on-disk’ scheme.

Despite these features, all four types of the CCF-reinforced composites were generally qualitatively similar in terms of the scratch track formation. This was an important pattern of these data, in contrast to the results of the tribological tests. Therefore, the obtained data were more important for describing local mechanical properties (by analogy with microhardness levels) than for explaining the tribological characteristics.

As a result, it was possible to conclude that the solid lubricating role of loaded microparticles was manifested to a minimum extent during the scratch test. In addition, the negative effect of PTFE on the PI-matrix structure formation also did not exert a noticeable impact on resistance to the indenter movement.

By way of discussion, a few relevant papers are cited below, where aspects of interphase adhesion, tribological load conditions, counterpart material and various solid lubricant fillers were studied. In [42] various tribological testing conditions were employed: (i) reciprocating pin-on-disk, (ii) rotating pin-on-disk, and (iii) thrust washer. It was shown that their variation resulted in changing friction coefficient as well as wear rate for the PEEK-PTFE composites. In [43] tribological behaviors of various polyimide (PI) composites at sliding over medium carbon steel and NiCrBSi were studied. For the PI composite filled with carbon fibers and graphite the carbon-based tribofilm formed on NiCrBSi surface resulted in reduction of friction- and wear. However, no lubricating tribofilm was formed on MCS35 surface. In doing so, chelation of polymeric molecular radicals with the metallic counterparts was revealed on the worn surfaces.

Various aspects of nanoscale structures of the transfer films of polyimide (PI) composites formed when slid against various metallic counterparts (including standard bearing steel, electroplated chromium coating, and stainless steel) were investigated in [44]. The fillers were: (i) short carbon fibers (20–45 μm in length and 7 μm in diameter); (ii) graphite flakes (particles with the size ≈ 4 μm); (iii) amorphous nano-silica (SiO_2_) with a diameter of 20 nm and (iv) h-BN nanoparticles with a diameter of 120 nm. When the composite slid over steel counterparts, obvious tribooxidation occurred and retarded material transfer. However, the composite showed a much-improved tribological performance when slid against Cr due to formation of a carbonaceous transfer film. Both kinds of nanoparticles significantly mitigated tribooxidation and greatly enhanced the tribological performance.

Aspects of increasing interphase adhesion between PI matric and carbonaceous fibers are of actuality as well. In [39], graphite fibers were prepared from PI-ones by doping varying contents of graphene oxide (GO) through a carbonization and graphitization process. Then, GO/polyamic acid was synthesized and used for preparing GO/PI fibres via dry-jet wet spinning. In [45] two dimensional (2D) MoS_2_ nanosheets were exfoliated by high shear mixing technique and further incorporated into PAA precursors to form the MoS_2_/PI nanocomposite films. It was shown that the mechanical and thermal properties of the prepared MoS_2_/PI nanocomposite films were significantly enhanced by adding a small amount of MoS_2_ nanosheets without any surface modification. Another paper of this research group [40] solved the problem of poor MoS_2_ nanoflowers dispersion in polyimide (PI). For doing so, they were accurately grafted onto the surface of hollow carbon nanofibers (HCNF). The MoS_2_-HCNF hybrid was utilized as homogeneous filler to enhance the tensile strength and lubricity of the PI-based composite.

Besides SCF, PI enforcement was attained through loading with CNT. In [46] the polyimide/multi-walled carbon nanotubes (MWNTs) nanocomposite films were prepared by mixing of poly(amic acid) (PAA) solution and MWNTs/DMAc suspension follow by mixture casting, evaporation and thermal imidization. With the incorporation of MWNTs, mechanical properties of the nanocomposite films were greatly improved due to the strong interfacial interaction between the modified MWNTs and the PI matrix. In similar research [47] polyimide/carbon nanotube (PI/CNT) nanocomposites with different proportions of CNT were fabricated by in situ process. It was shown that CNT effectively reduced friction and improved antiwear capacity of the nanocomposite because it increased the load capacity and mechanical strength of the CNT/PI. Another approach to form high performance MWCNT-reinforced polyimide nanocomposites is in situ polymerization [48]. It was shown that the nanocomposites with 0.50–0.75 wt. % MWCNTs had the most excellent thermomechanical and tensile properties at such low filler content.

Some papers attracted synergy concept for the interpretation of tribological testing results. It was revealed in [49] that the incorporation of individual fillers (i) graphite (Gr), (ii) carbon fiber (CF) and (iii) carbon nanotube (CNT) improved the wear resistance of polyimide (PI) under sea water lubrication, but did not decrease the friction coefficient. The combined incorporation of 10 vol. %Gr, 10 vol. %CF and 5 vol. %CNT was the most efficient in improving the anti-wear properties of PI. This suggested a synergetic effect among the three carbon series additions on improving the wear resistance of PI. In another paper on the topic [50], the effect of short carbon fiber (SCF), graphite (Gr) and nano-Si_3_N_4_ on the friction and wear behavior of polyimide (PI) composites were studied under “block-on-ring” tribological testing. It was shown that incorporation of SCF and Gr improved the friction-reducing and anti-wear abilities of the PI composites significantly. A synergistic effect was revealed for the combination of nano-Si_3_N_4_ and SCF and Gr, which provided the best tribological properties. In addition, the filled PI composites exhibited better tribological properties under higher PV product.

MoS_2_ particles are often loaded at development of antifriction composites based on high-strength high-temperature PEEK and PI plastics. In [51], the effect of MoS_2_ and WS_2_ nano-and microparticles on structure, hardness and wear resistance of PEEK-based composites were studied. A possibility of increasing hardness and wear resistance at sliding over 100Cr6 stainless steel was shown (surface roughness was R*_a_* = 0.030 μm; the counterpart sample was a flat-end cylinder with a diameter of 3 mm). It was reported that formation of a tribofilm is necessary to reduce the wear of all the composites; that was strongly promoted by the addition of nano-or microparticles of both the MoS_2_ and WS_2_ materials. In [52], thermoplastic polymide was reinforced by loading with molybdenum disulfide and its tribology performances was studied under dry and water lubrication. It was shown that the composites’ bending and compressive strength decreased compared to pure PI. In the dry sliding condition, the friction coefficient decreased when PI was loaded with MoS_2_ with lowest wear at MoS_2_ content of 10–15 wt. %. In doing so, the adhesive wear was a dominant mechanism. In [53], tribological properties of carbon fiber reinforced polyimide (PI) composites with different MoS_2_ content at sliding against GCr15 steel were studied under block-on-ring scheme. It was shown that the increasing MoS_2_ content up to 20 wt. % significantly improved the wear resistance and decreased the friction coefficient of CF reinforced PI composites. It was revealed that friction coefficient and wear rate of MoS_2_/SCF reinforced PI composites decreased with increasing applied load from 200 to 500 N. The decreasing of above tribological parameters also occurred with increasing sliding rate from 0.431 up to 0.862 m/s.

In doing so, the data of this research agree well with the above-described literature results.

Based on the results of the tribological tests, it was possible to conclude that loading of the CCF-reinforced PI-based composites with Gr and MoS_2_ solid lubricant fillers provided the consistently low wear rates in the entire investigated range of loads and sliding speeds. However, the role of the transfer film on the counterpart was leveled out in the tribological tests according to the ‘block-on-ring’ scheme (as opposed to the ‘pin-on-disk’ one). This was due to the higher counterpart roughness, lower specific pressure, and, probably, low temperatures in the tribological contacts. Therefore, the increase in wear resistance was substantially determined by the polymer composite structure (its uniformity and the presence/absence of defects).

For a more detailed study of these factors, further numerical experiments were carried out for the steel counterpart friction against the surfaces of the CCF-reinforced PI-based composites. Properties of the “PI/solid lubricant particles/CCF” composite were taken as for a medium with averaged characteristics.

## 5. Numerical Simulation of the Friction Process

**Figure 17:**
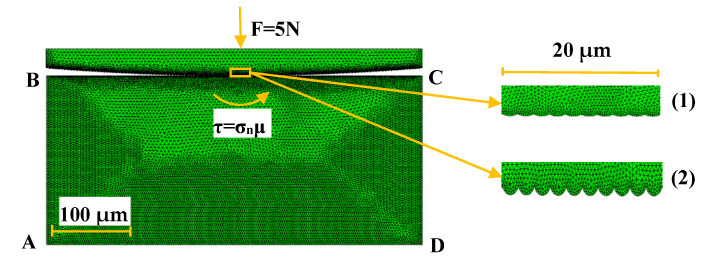
The computational domain with the applied finite element mesh and different counterpart roughness levels: (1) R*_a_* = 0.1 µm, (2) R*_a_* = 1 µm.

In order to study the influence of the counterpart roughness, the friction coefficient and the sample porosity on the polymer wear rates, the friction process was simulated according to the ‘block-on-ring’ scheme (Figure 17).

The contact problem of the solid mechanics was solved in a plane formulation at taking wear into account. Since the main loads (normal and tangential) acted in the same plane, the problem of a plane stress state was considered. To solve it, the finite element method with triangular simplex elements was applied. The problem statement and solution method were previously described in [54]. Finite elements were smaller (finer mesh) in the contact area (Figure 17; darker regions). The FE mesh size in the contact area was varied from 0.005 to 0.01 μm that depended on the size of asperities. On the ‘block-on-ring’ contact surface, the action of normal and tangential loads arising from friction was taken into account. The load was concentrated in the contact with a cylindrical counterpart (steel ring, located on top). In the plane case, a part of the ring (segment adjacent to the rectangular area of the specimen) was considered in the simulation (Figure 17). To simulate the counterpart roughness, the sample size was taken as 0.5 × 0.25 mm^2^ (ABCD region), while the metal counterpart diameter was 6 mm. The (i) computational domain size, (ii) counterpart diameter as well as (iii) its roughness were specified with the view of limitations on the FE mesh dimension. For doing so, pores of micron sizes were to be taken into account into explicit form. Both roughness values of 1.0 and 0.1 μm were considered (they differed from the experimental data due to computational restrictions on the FE mesh size). In the calculations, the shape of the initial contact surface of the polymer composite samples was taken to be smooth. When roughness and porosity were not taken into account the maximum Hertzian contact pressure was equal to 12.6 MPa (before start of the testing). When pores were not considered, the maximum Hertzian contact pressure was equal to 32 MPa at roughness value of 0.1 μm, while it decreased down to 16 MPa at roughness value of 1 μm. The sample properties were consistent with the PI/10CCF/10PTFE composite (Table 2). The loading diagram nonlinearity was considered (Figure 1, curve 3) and the friction coefficient was taken as 0.083.

The simulation was carried out using the method of successive loads (its values were set at each step). The total normal stress in the contact, multiplied by its area, was equivalent to the normal load. The tangential load was considered at the points of the contact on the sample surface according to the Coulomb’s law. At each load step, the following fracture criteria were checked for the sample: (1) the stress intensity reached the yield point at the contact boundary, (2) the excess of the maximum permissible tangential stress value. The fracture process was realized by removing mesh elements, in which the criterion was met, from the contact surface calculation. After that, a new contact boundary was formed, and the finite element mesh was rebuilt. The number of removed elements in one surface region could be any, as a result of which irregularities of different sizes appeared. For this reason, the dimensions of the elements depended on both sizes of the irregularities (asperities) and fulfillment of the mesh division uniformity condition. On the one hand, sizes of elements could not exceed the permissible value of the mutual overlap of the contact surfaces. On the other, they could not be larger than the counterpart roughness. So, it was taken as a parameter defining the overlap coefficient and the dimensions of the elements. Based on the convergence of the obtained results, the value of the mutual overlap of the surfaces was 0.01 µm for both roughness values of 1 µm and 0.1 µm.

Due to the simulation complexity, pores were taken in the form of inclusions with the minimum possible elastic modulus, namely, six orders of magnitude less than that for the PI/10CCF/10PTFE composite (Table 2). Their sizes were 3–5 µm with volume contents of 2%. Pores were randomly distributed over the computational domain. For inclusions (pores) located on the contact surface, the criterion of removal of elements was automatically fulfilled.

**Figure 18:**
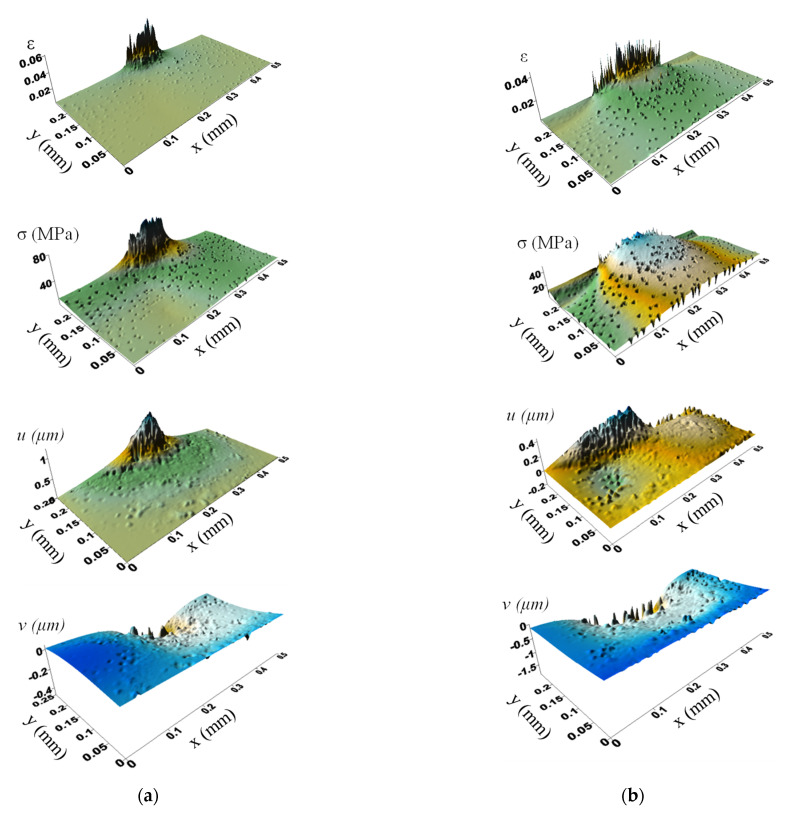
The surfaces of strains and stress intensities as well as displacements along the X (u) and Y (v) axis; pores are taken into account; the counterpart roughness of 0.1 μm (**a**) 1 μm (**b**).

Figure 18 presents the calculated surfaces of strains, stresses and displacements along the X and Y axes. At higher roughness value, the deformed region was evidently wider and deeper, while the stresses were decreased due to increasing the contact area. The normal load action had a predominant effect on displacements along the X axis, as there were positive and negative movements caused by the counterpart indentation. Displacements along the Y-axis were 3 times larger in the case of rougher counterpart, while the contact area was also much larger.

**Figure 19:**
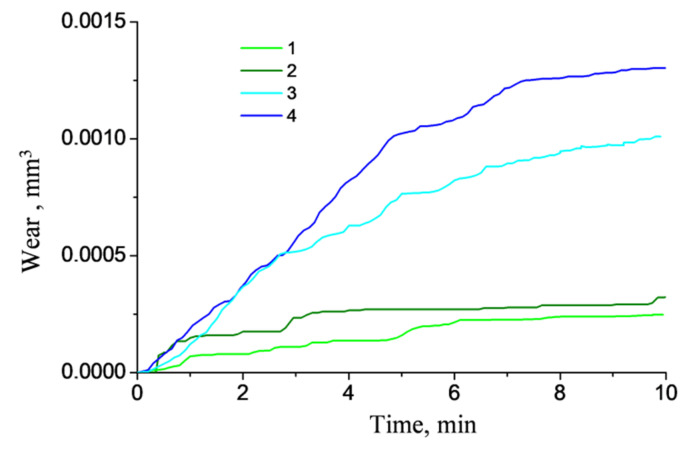
Wear rates of the PI/10CCF/10PTFE composite without pores (1, 3) and with them (2, 4) for different counterpart roughness: 0.1 μm (1, 2) and 1 μm (3, 4).

Figure 19 shows the wear kinetics for the PI/10CCF/10PTFE composites both with and without pores at different roughness counterparts. An increase in the roughness enhanced wear rate, especially in the presence of pores.

**Figure 20:**
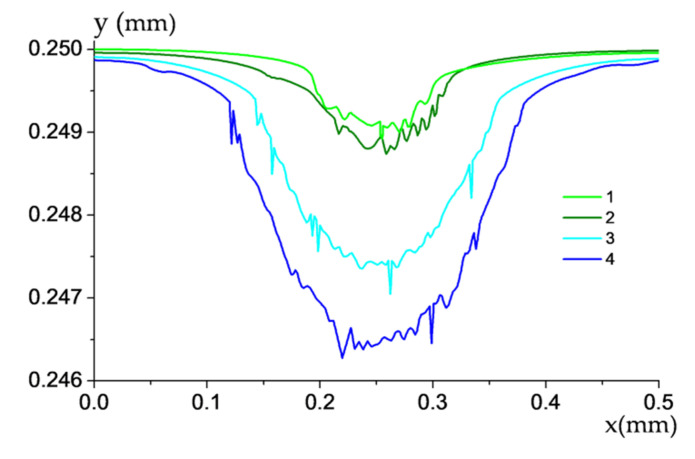
The wear track profiles of the PI/10CCF/10PTFE composite without pores (1, 3) and with them (2, 4) for different counterpart roughness; 0.1 μm (1, 2) and 1 μm (3, 4).

Figure 20 presents the wear track profiles drawn according to the parameters of the computational grid with removed elements for different counterpart roughness (after 10 min of sliding). They corresponded to the graphs shown in Figure 19. The deepest wear tracks (i.e., more intense wear) was characterized for the counterpart with greater roughness when it sliding over the porous composite. In addition, the level of roughness exerted a significantly greater effect on wear rate of the CCF-reinforced PI-based composites than porosity.

Hence, the effect of the counterpart roughness and the presence of microporosity in the polymer composites was evaluated in the numerical experiment simulated the ‘block-on-ring’ tribological test. The obtained results enabled to interpret the fact of the increase in wear rate for the PI/10CCF/10PTFE composite for the ‘block-on-ring’ test scheme compared to the ‘pin-on-disk’ one, considering the changed counterpart roughness and the PI/PTFE matrix porosity.

## 6. Conclusions

The structure, mechanical and tribological properties of the CCF-reinforced PI-based composites loaded with solid-lubricant commercially available fillers (PTFE, Gr, MoS_2_) were investigated under the metal- and ceramic-polymer contacts. It was shown that addition of CCF with the length of 2 mm in the amount of 10 wt. % increased the elastic modulus by 2.5 times and ultimate tensile strength by 1.5 times.

It was found that the scheme and loading conditions exerted the great effect on wear resistance of the composites. In the tribological tests according to the ‘pin-on-disk’ scheme, the PI/CCF/PTFE one possessed the highest wear resistance. In this case, wear rate decreased down to ~290 times for the metal-polymer tribological contact and to ~285 times for the ceramic-polymer one compared to those for neat PI. Loading with solid-lubricant fillers (Gr, MoS_2_) improved wear resistance of the CCF-reinforced composites only by 37 and 30 times (Gr), as well as by 7 and 3 times (MoS_2_) for the metal- and ceramic-polymer tribological contacts, respectively. This effect manifested itself under the smooth counterpart sliding conditions (roughness of less than 0.02 μm) on the ‘renewable’ polymer composite surface (without a ‘shortage’ of solid lubricant particles on the wear track surface) at the load of 5 N and the sliding speed of 0.3 m/s. The reason was the formation of the transfer film on the counterpart friction surface, as well as the similar one on the polymer composite (under both conditions of the metal- and ceramic-polymer tribological contacts). At the same time, the change in the friction coefficient and wear rate correlated well with each other.

In the tribological tests according to the ‘block-on-ring’ scheme on the rougher counterpart (R*_a_*~0.2 μm) in the studied range of loads (60–180 N) and sliding speeds (0.1–0.5 m/s), both Gr and MoS_2_ ensured high wear resistance of the three-component composites. The possibility of the transfer film formation was minimized, since the large-area counterpart slid over the ‘non-renewable’ surface of the polymer composite with a ‘shortage’ of solid lubricant particles. On the other hand, these particles served as reinforcing inclusions (except the PTFE). Under these conditions, the important aspect was the formation of the dense structure. The latter also took place in the CCF-reinforced PI-based composite without solid lubricant particles.

Finally, numerical simulation of the tribological test according to the ‘block-on-ring’ scheme was carried out. Within the implemented model, roughness exerted a significantly greater impact on the wear rate than the porosity. The obtained results enabled to interpret the experimentally revealed fact of increased wear rate for the PI/10CCF/10PTFE composite for the ‘block-on-ring’ test scheme compared to the ‘pin-on-disk’ one, considering the changed counterpart roughness and the polymer matrix porosity.

## Figures and Tables

**Table 1:** The compositions of the studied PI-based composites.

No.	Designation	Filler Content, vol. %	Filler Content, wt. %
1	PI	PI	-
2	PI/10CCF	8.3%CF	10%CF
3	PI/10CCF/10PTFE	8.3%CF+6.6%PTFE	10%CF+10%PTFE
4	PI/10CCF/10Gr	8.3%CF+6.6%Gr	10%CF+10%Gr
5	PI/10CCF/10MoS_2_	8.3%CF+3.0%MoS_2_	10%CF+10%MoS_2_
6	PI/10CCF/23MoS_2_	8.3%CF+6.6%MoS_2_	10%CF+23%MoS_2_

**Table 2:** The physical and mechanical properties of the PI-based composites.

No.	Filler Composition (wt. %)	Density *ρ*, (g/cm^3^)	Shore *D* Hardness	Elastic Modulus E (GPa)	Ultimate Tensile Strength σ_U_ (МPа)	Elongation at Break ε (%)
1	PI	1.37	80.2 ± 0.8	2.60 ± 0.69	110.7 ± 1.0	13 ± 0.7
2	PI/10CCF	1.42	80.6 ± 0.4	6.40 ± 0.33	152.1 ± 6.4	5.9 ± 0.3
3	PI/10CCF/10PTFE	1.44	77.5 ± 0.6	5.79 ± 0.45	115.9 ± 10.8	4.1 ± 0.3
4	PI/10CCF/10Gr	1.46	80.1 ± 0.3	6.35 ± 0.24	105.0 ± 3.9	2.7 ± 0.1
5	PI/10CCF/10MoS_2_	1.51	82.0 ± 0.3	6.06 ± 0.32	113.1 ± 9.1	3.0 ± 0.1
6	PI/10CCF/23MoS_2_	1.67	82.7 ± 0.3	7.56 ± 0.31	118.4 ± 11.9	2.5 ± 0.7

**Table 3:** The tribological characteristics of the PI-based composites (*P* = 5 N; *V* = 0.3 m/s; the ‘pin-on-disk’ scheme).

Composite	The Friction Coefficient ƒ		Wear Rate (10^−6^ mm^3^/N × m)	
	Metal Counterpart	Ceramic Counterpart	Metal Counterpart	Ceramic Counterpart
PI	0.388 ± 0.012	0.375 ± 0.008	134.16 ± 18.5	77.34 ± 2.25
PI/10CCF	0.304 ± 0.036	0.353 ± 0.023	27.86 ± 3.42	10.03 ± 2.99
PI/10CCF/10PTFE	0.083 ± 0.003	0.094 ± 0.007	0.46 ± 0.03	0.27 ± 0.02
PI/10CCF/10Gr	0.211 ± 0.020	0.226 ± 0.023	3.54 ± 0.22	2.60 ± 0.23
PI/10CCF/10MoS_2_	0.302 ± 0.008	0.295 ± 0.021	18.25 ± 1.64	23.71 ± 2.31
PI/10CCF/23MoS_2_	0.265 ± 0.048	0.262 ± 0.022	45.67 ± 4.02	43.66 ± 1.90

**Table 4:** The results of EDS analysis of the transfer films and debris on the steel counterpart surfaces in accordance with the labels in Figure 8.

Element	Spectrum 1 wt. %/at. %	Spectrum 2 wt. %/at. %	Spectrum 3 wt. %/at. %	Spectrum 4 wt. %/at. %	Spectrum 5 wt. %/at. %
PI/10CCF/10PTFE					
Cr	1.18/1.27	0.92/0.52	0.68/0.31	3.10/2.76	1.45/1.35
Fe	98.82/98.73	73.23/38.64	57.43/23.95	91.19/76.42	93.42/81.00
F	-	0.78/1.21	4.81/5.90	1.02/2.51	2.05/5.22
C	-	21.99/53.96	32.84/63.68	4.70/18.30	3.08/12.43
O	-	3.08/5.67	4.24/6.17	-	-
PI/10CCF/10Gr					
Cr	0.94/1.01	3.96/3.91	1.39/1.35	0.23/0.06	0.90/0.90
Fe	99.06/98.99	93.74/86.22	95.90/87.19	13.38/3.41	96.90/89.65
C	-	2.31/9.87	2.71/11.46	66.21/78.55	2.20/9.45
O	-	-	-	20.17/17.97	-
PI/10CCF/10MoS_2_					
Cr	1.34/1.31	0.98/0.97	-	-	
Fe	95.86/86.89	91.13/83.96	0.77/0.20	1.02/0.26	0.86/0.22
C	2.80/11.81	-	65.48/77.84	68.92/81.65	66.07/77.73
O	-	4.04/13.01	15.51/13.84	10.65/9.47	16.89/14.92
Mo	-	3.85/2.06	-	-	
S	-	-	18.24/8.12	19.41/8.61	16.18/7.13

**Table 5:** The results of EDS analysis in accordance with the labels in Figure 12.

Element	Spectrum 1 wt %/at. %	Spectrum 2 wt %/at. %	Spectrum 3 wt %/at. %	Spectrum 4 wt %/at. %	Spectrum 5 wt %/at. %
PI/10CCF					
C	5.67/13.54	42.11/59.81	-	-	-
O	28.93/51.6	28.44/30.32	30.68/52.86	30.56/57.87	47.32/71.13
Cr	0.73/0.40	0.20/0.07	-	0.56/0.33	-
Fe	64.67/34.46	29.25/9.8	69.32/47.14	68.88/41.8	52.68/28.87
PI/10CCF/10PTFE					
C	53.87/64.24	9.05/14.96	27.92/44.00	52.87/63.69	5.74/12.12
O	26.11/21.93	36.12/54.23	32.37/38.33	23.58/19.53	34.74/55.01
F	20.02/13.83	8.55/9.31	4.34/4.32	23.55/16.78	4.51/6.02
Cr	-	3.31/1.81	0.36/0.13	-	0.77/0.37
Fe	-	42.97/19.69	35.01/13.22	-	54.24/26.48
PI/10CCF/10Gr					
C	53.49/60.72	10.21/22.53	13.40/26.26	68.44/79.58	51.71/59.31
O	45.92/39.13	29.45/48.80	35.45/52.16	20.10/17.54	47.31/40.45
Cr	0.16/0.04	0.65/0.33	0.57/0.26	0.28/0.08	0.24/0.06
Fe	0.43/0.11	59.69/28.33	50.58/21.32	11.18/2.80	0.74/0.18
PI/10CCF/10MoS_2_					
C	39.57/57.27	6.35/15.91	22.42/39.41	14.83/29.93	22.22/42.34
O	30.06/32.67	24.90/46.86	33.10/43.68	30.16/45.68	24.15/34.55
S	6.68/3.62	4.20/3.93	3.81/2.51	6.45/4.88	8.40/6.00
Cr	0.09/0.03	0.97/0.56	0.49/0.20	0.41/0.19	0.57/0.25
Fe	20.67/5.87	59.34/31.41	37.20/13.54	44.09/18.3	39.04/15,52
Mo	2.93/0.54	4.24/1.33	2.98/0.66	4.05/1.02	5.62/1.34

## Data Availability

The data presented in this study are available on request from the corresponding author.
